# Factors influencing self-reported perceived health among United States adults with arthritis: A cross-sectional study

**DOI:** 10.1097/MD.0000000000042286

**Published:** 2025-04-25

**Authors:** David R. Axon, Taylor Butler

**Affiliations:** a Department of Pharmacy Practice and Science, R. Ken Coit College of Pharmacy, The University of Arizona, Tucson, AZ

**Keywords:** arthritis, health status

## Abstract

Limited knowledge exists of how various characteristics contribute to perceived health among the millions of United States (U.S.) adults with arthritis. This study explored the relationship between various factors and the perceived health of U.S. adults with arthritis. U.S. adults (18 years or older) alive (throughout 2021) and self-reporting a diagnosis of arthritis in the 2021 Medical Expenditure Panel Survey (MEPS) data were included in this study. Multivariable logistic regression was applied to determine the associations between independent variables (age, sex, race, ethnicity, education, employment, marriage, income, insurance, pain, chronic conditions, functional limitations, physical activity, smoking, region) and good versus poor perceived health, accounting for the complex survey design and weighting to reflect the U.S. population. Statistical significance was set at *P* < .05. The study included 5108 eligible participants (74.4% good health, 25.6% poor health). Factors associated with good perceived health included age ≥ 70 years (odds ratio [OR] = 3.5, 95% confidence interval [CI] = 2.0–6.2) and age 60 to 69 years (OR = 2.0, 95% CI = 1.2–3.5) versus age 18 to 39, females versus males (OR = 1.2, 95% CI = 1.0–1.5), white versus multiple races (OR = 2.0, 95% CI = 1.2–3.3), employed versus unemployed (OR = 2.0, 95% CI = 1.5–2.7), high versus low income (OR = 1.3, 95% CI = 1.0–1.7), little/moderate versus quite a bit/extreme pain (OR = 3.7, 95% CI = 3.0–4.7), <2 versus ≥ 2 chronic conditions (OR = 1.4, 95% CI = 1.1–1.8), no functional limitations versus functional limitations (OR = 2.0, 95% CI = 1.6–2.5), and regular versus no regular physical activity (OR = 2.2, 95% CI = 1.7–2.9). This study identified several factors that could be targeted to better manage and prevent arthritis in U.S. adults and improve perceived health.

## 1. Introduction

Arthritis is a common and incurable diagnosis encompassing a variety of bone and joint inflammatory disorders.^[1]^ It has a costly burden on millions of Americans and the United States (U.S.) healthcare system.^[1]^ There are over 100 different types of arthritis and arthritis-related diseases, ranging from juvenile arthritis to gout and ankylosing spondylitis.^[2]^ However, the most prevalent forms of arthritis are osteoarthritis (OA) and rheumatoid arthritis (RA).^[2]^ Together, these affect over 33.8 million Americans.^[2]^ OA is characterized by the irreversible disintegration of cartilage connecting bones and joints.^[3]^ This commonly leaves a debilitatingly painful stiffness that can impair quality of life and keep affected individuals out of work.^[3]^ In 2017, the in-hospital cost-burden of OA was the second highest among all U.S. healthcare expenditures at $19.9 billion dollars, second only behind sepsis.^[4]^ In 2013, the total healthcare costs and losses related to arthritis (both inpatient and outpatient) reached over $300 billion.^[5]^ The second most common form of arthritis, RA, is a chronic autoimmune disorder.^[6]^ With RA, the body mistakenly attacks the healthy tissue cells surrounding the bones.^[6]^ This leads to painful inflammation throughout the body.^[6]^ In turn, this can often affect other parts of the body including (but not limited to) the blood vessels, heart, kidneys, eyes, and nerves during periods of flare-up.^[6]^

Arthritis, of any type, is the most commonly reported disability in the U.S.^[5]^ Individuals are at greater risk of experiencing falls and fractures due to the pathology of the disease and the side effects of the medications required to treat it.^[5]^ Aside from the evident effects of arthritic conditions on bone health and mobility, there are other less obvious consequences of arthritis. It is conceivable that many treatment plans consist of medications to improve a persons’ mental health. This is due to the combination of severe, sometimes chronic pain, and the inability to be as active as once accustomed.^[7,8]^ Data suggests that there may even be a biological relationship between the increased release of peripheral cytokines and neurologic inflammation seen in RA with depression.^[9]^ In fact, some studies suggest that half of all patients with RA experience some type of depression.^[10]^ In addition, 26.2% of patients with any form of arthritis experience frequent anxiety and depression.^[10]^ This is especially concerning because depression has been linked to lowered efficacy of typical treatment medications and more frequent flare-ups.^[11]^ There is also a concerning relationship between RA and heart disease due to potential inflammation of the blood vessels causing an increased risk of stroke or myocardial infarction.^[12]^ The health concerns associated with at least one major form of arthritis makes activities of daily living more difficult for patients who are already living with a non-curable, progressive disease.

Several studies have been published related to the health effects of arthritis and potential genes that may be involved in its origination. Yet, there remains a gap in knowledge regarding how the personal attributes, lifestyle, and environmental factors of a person with arthritis correlates with their health status. To this end, this study seeks to determine if there is any significant relationship between perceived health among U.S. adults with arthritis based upon factors such as their age, race, education, employment status, marital status, income, level of pain, degree of functionality, physical activity, smoking status, and geographical region. While some studies may have included some of these variables, this study provides a more complete understanding of these variables among a large representative sample of the U.S. population.

## 2. Methods

The data for this study were collected from the 2021 Medical Expenditure Panel Survey (MEPS). MEPS is sponsored by the Agency for Healthcare Research and Quality as a part of the U.S. Department of Health and Human Services. The annual MEPS report consolidates extensive survey data from 3 sectors – Household, Healthcare Providers, and Insurers. This allows researchers to gain insights into the health status and characteristics, costs of care, and coverage of a nationally representative subset of the American population.^[13,14]^

To obtain information at the individual and family level, selected individuals who completed the previous year’s census requirements were asked to participate in several interviews over a two-year period. These interviews collected data on household demographics, health status, insurance coverage or payment methods, and costs of care.^[13]^ The MEPS data file also includes information from a wide variety of public and private employers to assess the health insurance options available to their employees, including associated costs and premiums.^[15]^ The final piece of the MEPS dataset interweaves information gathered from medical providers (i.e., physicians, pharmacists, home health organizations, etc.). These providers are directly associated with the individuals in the household survey and corroborates the responses of surveyed individuals.^[15]^

To meet the eligibility requirements for this cross-sectional study, U.S. participants had to be at least 18 years old, self-report a diagnosis of arthritis, and remain alive throughout the 2021 study period. The dependent variable assessed perceived health using a MEPS item that asked about perceived health with response options ranging from excellent, very good, good, fair, or poor. Responses were dichotomized as good (excellent/very good/good) or poor (fair/poor) perceived health. Independent variables included participant age (18–39, 40–49, 50–59, 60–69, ≥70), sex (female, male), race (white, black, American Indian/Alaska Native, Asian/Native Hawaiian/Pacific Islander, multiple races), ethnicity (Hispanic, non-Hispanic), education (high school or less, more than high school), employment (employed, unemployed), marital status (married, not married), income (mid-high, low), insurance coverage (private, public, none), pain (little/moderate, quite a bit/extreme), chronic conditions (less than 2, 2 or more), functional limitations (no, yes), regular physical activity (yes, no), current smoking status (yes, no), and geographic region (Northeast, Midwest, South, West).

The data analysis for this study was conducted using SAS (SAS Institute Inc., Cary). A chi-squared analysis was employed to examine each independent variable in relation to self-reported perceived health (categorized as either good or poor). A multivariable logistic regression model was created to assess statistical significance between variables. The analysis accounted for the complex survey design using strata and cluster MEPS variables. An alpha value of 0.05 was chosen a priori, and the MEPS weighting variable was applied to obtain nationally representative estimates. Results were reported using odds ratios (OR) and confidence intervals (CI). The assumptions of the logistic regression analysis were assessed, and the c-statistic and Wald statistic for the regression model were reviewed. This study received approval from the University of Arizona Institutional Review Board (approval number: 00004719, date: 06/06/2024).

## 3. Results

The 2021 MEPS dataset included valid responses from 28,336 participants, out of which 5108 met the study eligibility criteria. Among these, 3708 participants reported good perceived health, while 1400 indicated poor perceived health. The eligible responses were weighted to reflect the U.S. population (N = 64,186,897). Approximately 74.4% of respondents reported good perceived health (95% CI = 72.9–76.0), with 25.6% reporting poor perceived health (95% CI = 24.0–27.1).

Overall, participants in both perceived health groups (good perceived health and poor perceived health) were predominantly older, female, white, non-Hispanic, with education beyond high school, unemployed, married, had mid-high income, private insurance, little/moderate pain, at least 2 chronic conditions, no functional limitations, do not engage in regular physical activity, do not smoke, and reside in the southern region of the U.S. All variables showed statistically significant differences between groups (*P* < .05), except for participant sex and region (Table 1).

**Table 1:** Characteristics of United States adults with arthritis in the weighted population.

Variable	Good health N = 3708 % [95% CI]	Poor health N = 1400 % [95% CI]	Total N = 5108 % [95% CI]	*P* value
Age				.0049
≥70 years	38.3 [36.1, 40.5]	31.4 [28.2, 34.6]	36.5 [34.7, 38.4]	
60–69 years	27.8 [25.9, 29.7]	28.5 [25.7, 31.4]	28.0 [26.3, 29.7]	
50–59 years	18.3 [16.4, 20.2]	22.8 [19.7, 25.9]	19.4 [17.8, 21.1]	
40–49 years	9.5 [8.0, 11.0]	9.4 [7.1, 11.8]	9.5 [8.1, 10.8]	
18–39 years	6.1 [4.8, 7.4]	7.9 [5.8, 9.9]	6.6 [5.4, 7.7]	
Sex				.9819
Female	60.6 [58.8, 62.5]	60.6 [57.4, 63.8]	60.6 [59.1, 62.2]	
Male	39.4 [37.5, 41.2]	39.4 [36.2, 42.6]	39.4 [37.8, 40.9]	
Race				<.0001
White	82.7 [80.7, 84.7]	74.9 [71.2, 78.5]	80.7 [78.7, 82.8]	
Black	11.2 [9.5, 12.9]	16.3 [13.3, 19.2]	12.5 [10.8, 14.3]	
American Indian/Alaska Native	0.6 [0.3, 0.9]	0.9 [0.3, 1.4]	0.7 [0.4, 1.0]	
Asian/Native Hawaiian/Pacific Islander	3.4 [2.4, 4.4]	3.6 [1.5, 5.7]	3.5 [2.4, 4.5]	
Multiple races	2.0 [1.4, 2.6]	4.4 [2.8, 6.1]	2.6 [1.9, 3.3]	
Hispanic				.0070
Yes	8.5 [6.9, 10.0]	11.2 [8.9, 13.6]	9.2 [7.6, 10.7]	
No	91.5 [90.0, 93.1]	88.8 [86.4, 91.1]	90.8 [89.3, 92.4]	
Education				<.0001
≤High school	38.2 [36.0, 40.3]	53.3 [49.6, 57.0]	42.0 [40.1, 43.9]	
>High school	61.8 [59.7, 64.0]	46.7 [43.0, 50.4]	58.0 [56.1, 59.9]	
Employment				<.0001
Employed	46.2 [44.0, 48.4]	25.7 [22.4, 29.0]	41.0 [39.1, 42.8]	
Unemployed	53.8 [51.6, 56.0]	74.3 [71.0, 77.6]	59.0 [57.2, 60.9]	
Marriage				<.0001
Married	56.3 [54.2, 58.4]	45.1 [41.1, 49.1]	53.4 [51.4, 55.4]	
Not married	43.7 [41.6, 45.8]	54.9 [50.9, 58.9]	46.6 [44.6, 48.6]	
Income				<.0001
Mid-high	75.7 [73.7, 77.6]	50.7 [47.1, 54.3]	69.3 [67.3, 71.3]	
Low	24.3 [22.4, 26.3]	49.3 [45.7, 52.9]	30.7 [28.7, 32.7]	
Insurance				<.0001
Private	63.2 [60.9, 65.5]	43.9 [40.4, 47.4]	58.3 [56.2, 60.3]	
Public	34.5 [32.4, 36.6]	54.1 [50.6, 57.6]	39.5 [37.6, 41.4]	
None	2.3 [1.5, 3.1]	2.0 [0.8, 3.1]	2.2 [1.5, 3.0]	
Pain				<.0001
Little/moderate	83.9 [82.0, 85.8]	44.1 [40.0, 48.1]	70.7 [68.5, 72.9]	
Quite a bit/extreme	16.1 [14.2, 18.0]	55.9 [51.9, 60.0]	29.3 [27.1, 31.5]	
Chronic conditions				<.0001
<2	39.8 [37.6, 41.9]	25.2 [22.2, 28.3]	36.1 [34.2, 37.9]	
≥2	60.2 [58.1, 62.4]	74.8 [71.7, 77.8]	63.9 [62.1, 65.8]	
Functional limitation				<.0001
No	75.1 [73.3, 76.9]	39.8 [35.9, 43.8]	66.1 [64.2, 68.0]	
Yes	24.9 [23.1, 26.7]	60.2 [56.2, 64.1]	33.9 [32.0, 35.8]	
Regular physical activity				<.0001
Yes	53.7 [51.3, 56.1]	26.1 [22.9, 29.4]	46.6 [44.7, 48.6]	
No	46.3 [43.9, 48.7]	73.9 [70.6, 77.1]	53.4 [51.4, 55.3]	
Current smoker				<.0001
Yes	11.7 [10.1, 13.2]	19.5 [17.0, 22.1]	13.7 [12.3, 15.0]	
No	88.3 [86.8, 89.9]	80.5 [77.9, 83.0]	86.3 [85.0, 87.7]	
Region				.2350
Northeast	17.4 [14.2, 20.6]	16.4 [13.0, 19.8]	17.1 [14.1, 20.2]	
Midwest	22.3 [18.9, 25.8]	22.0 [17.7, 26.3]	22.2 [18.9, 25.6]	
South	39.3 [35.2, 43.4]	43.0 [37.9, 48.2]	40.3 [36.3, 44.3]	
West	21.0 [17.4, 24.5]	18.6 [14.8, 22.3]	20.4 [17.0, 23.7]	

In the multivariable logistic regression model, participants were associated with higher odds of reporting good perceived health if they were aged ≥70 years or 60 to 69 years versus 18 to 39 years, female versus male, white versus multiple races, employed versus unemployed, having a mid-high versus low income, reporting little/moderate versus quite a bit/extreme pain, having fewer than 2 versus 2 or more chronic conditions, having no functional limitations, and engaging in regular physical activity. There was no significant association between the remaining age groups, race categories, ethnicity, education, marriage, insurance coverage, smoking status, and geographic region (Table 2).

**Table 2:** Associations of good (versus poor) perceived health among United States adults with arthritis.

Variable	Odds ratio [95% confidence interval]
Age ≥ 70 years vs 18–39 years	3.5 [2.0, 6.2]
Age 60–69 years vs 18–39 years	2.0 [1.2, 3.5]
Age 50–59 years vs 18–39 years	1.3 [0.7, 2.2]
Age 40–49 years vs 18–39 years	0.9 [0.5, 1.8]
Sex Female vs Male	1.2 [1.0, 1.5]
Race White vs Multiple	2.0 [1.2, 3.3]
Race Black vs Multiple	1.4 [0.8, 2.5]
Race American Indian/Alaska Native vs Multiple	1.7 [0.7, 4.0]
Race Asian/Native Hawaiian/Pacific Islander vs Multiple	1.6 [0.6, 4.1]
Hispanic yes vs no	0.7 [0.5, 1.0]
Education ≤ High school vs >High school	0.9 [0.7, 1.1]
Employment Employed vs Unemployed	2.0 [1.5, 2.7]
Marriage Married vs Not married	1.1 [0.9, 1.4]
Income Mid-high vs Low	1.3 [1.0, 1.7]
Insurance Private vs none	1.0 [0.6, 1.9]
Insurance Public vs none	0.9 [0.5, 1.6]
Pain Little/moderate vs Quite a bit/extreme	3.7 [3.0, 4.7]
Chronic conditions < 2 vs ≥2	1.4 [1.1, 1.8]
Functional limitation no vs yes	2.0 [1.6, 2.5]
Regular physical activity yes vs no	2.2 [1.7, 2.9]
Current smoker yes vs no	0.9 [0.7, 1.2]
Region Northeast vs West	0.9 [0.7, 1.3]
Region Midwest vs West	1.1 [0.7, 1.6]
Region South vs West	1.1 [0.8, 1.6]

## 4. Discussion

This study identified several factors associated with good self-reported perceived health among U.S. adults with arthritis in the multivariable logistic regression model. The older age groups (≥70 years and 60–69 years) were associated with higher odds of reporting good perceived health relative to younger adults (18–39 years). Yet, there was no statistical association for middle aged adults (40–49 years, 50–59 years). It has already been reported that the prevalence of arthritis increases with age. For instance, data from the 2019 to 2021 National Health Interview Survey found that over 88% of arthritis cases were among U.S. adults aged ≥ 45 years.^[16]^ However, it was interesting that the older adults with arthritis reported higher odds of good health relative to younger adults in the current study. There is little contemporary literature available to explain this finding, which warrants further investigation. One possible explanation that we offer is older adults may be more accepting of having a chronic condition (such as arthritis) that impacts their health. Whereas, younger adults would perhaps expect to be healthier and not have a chronic condition such as arthritis. As a result, older adults may not perceive their health as poorly as younger individuals with the same condition. Furthermore, research indicates that older adults are more likely to use healthcare services.^[17]^ Thus, it is possible that older age adults are engaging with healthcare professionals to manage their condition better than middle aged adults. Additionally, aging is often associated with decreased mobility and a more sedentary lifestyle, which could limit the exacerbation of pain symptoms. Previous research by Booker et al^[18]^ and Hawker et al^[19]^ suggests that physical activity may worsen pain in individuals with osteoarthritis. This makes less-active individuals less likely to experience painful symptoms. Understanding these dynamics can help inform targeted interventions to improve health perceptions and management strategies across different age groups.

The current study found female adults were associated with higher odds of reporting good perceived health compared to male adults in the multivariable logistic regression analysis. However, in the descriptive analysis, no difference was found in the proportion of females and males reporting good versus poor perceived health. This differentiation may be accounted for by the adjustment of other variables that occurs in a multivariable regression model. Female sex has increased susceptibility to the development of OA relative to male sex,^[20]^ with females accounting for 60% of OA cases globally.^[21]^ One review identified several factors that contributed to the higher prevalence of OA among females.^[22]^ These factors included hormonal, cellular molecular, genetic, anatomical and biomechanical, obesity, and bone density.^[22]^ Furthermore, data from the 2021 Global Burden of Disease study found that females have a higher burden of morbidity-driven diseases compared to males.^[23]^ Another study by Tschon et al^[24]^ found that women are at greater risk of developing osteoarthritis and tend to experience more severe arthritis-related pain compared to men. However, the same study also indicated that women are more likely to utilize healthcare resources and undergo procedures.^[24]^ This may explain their higher likelihood of reporting better health status than men.^[24]^ Further research is warranted to explain the association of health status by sex among this population.

Considering the findings related to race in the current study, those of white race were associated with higher odds of reporting good perceived health compared to those of multiple races. A recent review by Yip et al^[25]^ found that most RA research is conducted on primarily white patients. The current research therefore offers value by assessing a nationally representative analysis of US adults with arthritis, to help establish associations between a larger variety of racial groups and perceived health status. The review by Yip et al^[25]^ also indicated that beyond biological factors, differences in socioeconomic status, therapeutic prescriptions, and healthcare access must be addressed to improve health equity for patients with RA. Research by Navarro-Millán et al^[26]^ has shown that African-Americans with RA are less likely to use biologic disease-modifying antirheumatic drugs – a cornerstone of treatment – compared to Caucasians. A similar study by Kerr et al^[27]^ comparing large cohorts of RA patients found that Caucasians were 1.7 times more likely to receive biologic treatments than non-Caucasians. This potentially explains why white respondents in this study were more likely to report better health as a result of their treatment. Ethnicity was not associated with perceived health in the current study. Yet, other studies that have reported poorer functional status and more severe disease levels among Hispanic and African-American individuals compared to white, non-Hispanic patients. This potentially highlights racial and ethnic disparities within the healthcare system.^[28,29]^ These disparities underscore the critical need for targeted interventions and outreach efforts that ensure equal access to healthcare resources and treatments for all individuals, regardless of age, sex, gender, race, or ethnicity. In future research, statistical interaction terms could be created and incorporated into the regression model. This may help reveal any potential interactions effects that may enhance our understanding of these relationships.

Employment status (employed vs non-employed) and income level (mid-high income vs low income) were associated with higher odds of reporting good perceived health in the current study. This is a logical finding, given that those who are employed are likely younger and not retired due to older age or poor health, and they are able to access healthcare services through workplace health insurance policies. Higher incomes also support the maintenance of a healthier lifestyle and access to healthcare services. These findings are in line with previous research by Silver et al,^[30]^ and Chetty et al,^[31]^ which linked unemployment with poorer health outcomes and higher income with longer life expectancy and improved health outcomes. The current study’s results align with these trends, as higher income levels are associated with lower rates of arthritis, according to recent data from the Centers for Disease Control and Prevention (CDC).^[32]^ These findings suggest that employment and income may play more critical roles in shaping self-reported health outcomes among individuals with arthritis. Therefore, prioritizing employment and income disparities should be a key focus for public health initiatives. Addressing these factors could lead to improved health outcomes and a better quality of life for individuals affected by arthritis.

Lower pain level and not having a functional limitation were both associated with higher odds of reporting good perceived health in the current study. Previous studies by Lee^[33]^ and Krustev et al^[34]^ have underscored the significance of pain as a key indicator of poor health among individuals with arthritis, as pain is often the primary reason for seeking medical care. Given that the primary objective of arthritis treatment is to prevent and alleviate musculoskeletal pain, it is unsurprising that individuals reporting higher pain levels in this study also indicated poorer health status.^[33]^ Capturing data on pain is always challenging, and this is particularly the case in individuals with arthritis. There is a need to use valid instruments to consistently and accurately capture the experience of pain among patients with arthritis to better measure its impact.^[35]^ Using a pain intensity numeric rating scale and the Patient-Reported Outcomes Measurement Information system pain interference test, one recent study found that pain centralization was associated with more intense pain among patients with RA.^[36]^

Research has indicated that pain has a direct contribution on functional limitations among people with arthritis.^[37,38]^ Previous studies over several years have reported a considerable impact of arthritis on various types of limitations, including restricted mobility, climbing stairs, and slower walking.^[39,40]^ It is therefore expected that individuals with self-reported arthritis and functional limitations would report poorer health status, particularly since the primary causes of functional limitations in those with RA are joint deformity, pain, and inflammation.^[41]^ These functional limitations can significantly impact a patient’s independence and quality of life, thus influencing their overall health perception. This highlights the urgent need for comprehensive pain management strategies and interventions that address both arthritis symptoms and coexisting conditions for individuals living with arthritis. Moving forward, there is a need for further drug development research to identify treatments that can better help alleviate the symptoms, limitations, and pain associated with arthritis.^[35]^

Having fewer chronic conditions was associated with higher odds of reporting good perceived health in the current study. This is an expected finding, given that healthier people tend to have fewer comorbidities. Specifically, among people with arthritis, research has demonstrated that coexisting chronic conditions are prevalent and that the symptoms of uncontrolled arthritis (e.g., pain, swelling, stress, loss of motion) can exacerbate these conditions, contributing to poorer overall health.^[19,42]^ This finding concurs with those found among other populations in the U.S. For instance, one MEPS study found that arthritis was the second most prevalent chronic condition among a sample of U.S. adults with some degree of pain.^[43]^ Meanwhile, another MEPS study found RA was the third most prevalent comorbidity among U.S. adults with hypertension.^[44]^

Doing regular physical exercise was associated with higher odds of reporting good perceived health in the current study. Again, this is an expected finding, given that a large body of evidence has consistently highlighted the positive effects of exercise on overall health and arthritis management.^[45,46]^ Exercise is a well-established, modifiable risk factor that can significantly improve joint mobility and enhance cardiovascular health.^[47]^ Importantly, it is one of the most accessible lifestyle interventions that individuals can adopt to improve their quality of life. However, it is crucial to recognize that many individuals with arthritis may struggle with pain and reduced mobility, which can limit their ability to engage in physical activity.^[48]^ This creates a barrier to experiencing the full benefits of exercise. Addressing these barriers through tailored exercise programs that accommodate pain and physical limitations is essential to ensuring that this population can fully benefit from physical activity.

The current study also observed that marital status, education level, insurance coverage, smoking status, and geographic region were not associated with perceived health among U.S. adults with arthritis. When considering marital status, there is little evidence to suggest there should be an association with health status in this population, and thus this finding is perhaps unsurprising and not a primary focus of further attention. However, the lack of association for some of the other variables, such as education status, is more interesting. While the current study did not observe an association between education level and health status, Pincus and Callahan found that individuals with RA who had more years of formal education were more likely to have longer lifespans and experience significantly lower rates of deterioration and mortality compared to those with less formal education.^[49]^ Another study established a causal relationship between higher educational attainment and a reduced incidence of RA development.^[50]^ The findings from the literature may be attributed to the increased likelihood that individuals with higher education levels understand the significant impact of a healthy lifestyle on physical health. Given that certain forms of arthritis may be preventable through healthy dietary practices and weight management, it is plausible that individuals with higher education levels leverage this knowledge to prevent or delay these conditions.^[51]^ Using this knowledge, policymakers should prioritize initiatives that enhance educational opportunities and promote health literacy to equip individuals with the tools they need to make informed decisions about their health.

The lack of statistical association between insurance status and perceived health status in this population of U.S. adults with arthritis was also noteworthy. One might expect individuals with health insurance to be associated with higher odds of reporting good health, since the provision of health insurance can help facilitate access to healthcare. However, there may be additional factors involved among U.S. adults with arthritis. For example, having health insurance may not be that helpful if meaningful treatments are not available for patients with arthritis. Extensive data indicates that having insurance, whether private or public, is correlated with improved health outcomes.^[52]^ The current study’s finding may be attributed to the observation that uninsured individuals utilize healthcare services less frequently than their insured counterparts, as reported by Taylor et al.^[53]^ This may be an area for further study, as understanding the implications of insurance status on health outcomes is crucial for improving access to care for these individuals.

Furthermore, smoking was not associated with health status in the current study. This finding is somewhat surprising given that smoking causes poor health regardless of comorbid conditions. It is further surprising due to the established role of smoking as a risk factor for the development of arthritis, particularly rheumatoid arthritis.^[54]^ While smoking is well known to negatively impact overall health, contributing to increased inflammation, cardiovascular disease, and impaired immune function, the absence of an association in this analysis may reflect complexities specific to this population of U.S. adults with arthritis. Further research is warranted to better understand the nuanced interactions between smoking and health outcomes in individuals with arthritis, and why its expected negative impact was not observed in this cohort.

Finally, there was no association with perceived health detected based on geographic region in the U.S. However, the broad categorization of the 4 large census regions may limit the ability to identify more nuanced regional differences. It is possible that smaller geographic areas, such as states or zip codes, could reveal patterns that are often overlooked by the larger regional divisions. Investigating the association of smaller unit areas and perceived health among this population could be considered in future work.

A key strength of this study is its large sample size and the inclusion of a nationally representative cohort, encompassing individuals from diverse backgrounds, ages, ethnicities, and educational statuses. Another strength of this study is the use of data from employers and certain respondents’ healthcare providers by the MEPS to verify survey responses, providing a more accurate depiction of Americans’ health status. Despite these strengths, the study has limitations due to its cross-sectional and self-reporting nature, as individuals’ responses may only reflect their condition at a specific moment. It is also plausible that participants exhibited recall bias or responded in a manner they believed was expected of them. There may also be potential statistical interactions between some of the variables in this study. Future studies should consider longitudinal approaches, following individuals throughout their arthritis diagnosis to observe significant changes over time, and incorporating statistical interaction terms into the regression model to reveal any potential synergistic interactions between variables. Given that respondents’ self-reported perceived health was dichotomized into 2 categories (good vs poor), future researchers might benefit from differentiating and analyzing each possible response separately.

## 5. Conclusion

Overall, this study offers valuable and contemporary insights into the perceived health of U.S. adults with arthritis by incorporating a range of variables and traits that have not often been analyzed together. The findings of this study demonstrate that a diverse set of variables are associated with perceived health and should be considered in future assessments to improve the health of Americans with arthritis. Future research should adopt a more holistic view of patient variables, as these often-overlooked factors may play a more significant role than previously understood.

## Author contributions

**Conceptualization:** David R. Axon, Taylor Butler.

**Data curation:** David R. Axon.

**Formal analysis:** David R. Axon.

**Funding acquisition:** David R. Axon.

**Investigation:** David R. Axon, Taylor Butler.

**Methodology:** David R. Axon, Taylor Butler.

**Project administration:** David R. Axon.

**Resources:** David R. Axon.

**Software:** David R. Axon.

**Supervision:** David R. Axon.

**Validation:** David R. Axon.

**Visualization:** David R. Axon, Taylor Butler.

**Writing – original draft:** David R. Axon, Taylor Butler.

**Writing – review & editing:** David R. Axon, Taylor Butler.
